# An Update on the Relationship of SARS-CoV-2 and Male Reproduction

**DOI:** 10.3389/fendo.2021.788321

**Published:** 2021-11-23

**Authors:** Juncen Guo, Kai Sheng, Sixian Wu, Hanxiao Chen, Wenming Xu

**Affiliations:** ^1^ Sichuan University-The Chinese University of Hong Kong (SCU-CUHK) Joint Laboratory for Reproductive Medicine, Key Laboratory of Obstetric, Gynaecologic and Paediatric Diseases and Birth Defects of Ministry of Education, West China Second University Hospital, Sichuan University, Chengdu, China; ^2^ Reproductive Endocrinology and Regulation Laboratory, Department of Obstetric and Gynaecologic, West China Second University Hospital, Sichuan University, Chengdu, China; ^3^ Department of Orthopedic Surgery, Shriners Hospital for Children, Montreal, QC, Canada; ^4^ Orthopaedic Research Laboratory, Department of Orthopedic Surgery, McGill University, Montreal, QC, Canada

**Keywords:** COVID-19, male reproduction, angiotensin-converting enzyme 2, erectile dysfunction, drug toxicity

## Abstract

Since the outbreak of the COVID-19, up to now, infection cases have been continuously rising to over 200 million around the world. Male bias in morbidity and mortality has emerged in the COVID-19 pandemic. The infection of SARS-CoV-2 has been reported to cause the impairment of multiple organs that highly express the viral receptor angiotensin-converting enzyme 2 (ACE2), including lung, kidney, and testis. Adverse effects on the male reproductive system, such as infertility and sexual dysfunction, have been associated with COVID-19. This causes a rising concern among couples intending to have a conception or who need assisted reproduction. To date, a body of studies explored the impact of SARS-CoV-2 on male reproduction from different aspects. This review aims to provide a panoramic view to understand the effect of the virus on male reproduction and a new perspective of further research for reproductive clinicians and scientists.

## Introduction

At the end of 2019, a novel coronavirus, named SARS-CoV-2, was found in patients with severe pneumonia and has become a worldwide pandemic with a rapidly growing number of infection cases up to now. SARS-CoV-2 and SARS-CoV-1 belong to the same coronavirus subfamily named the beta coronaviruses (1, 2). SARS-CoV-2 and SARS-CoV-1 share the same receptors named ACE2 residing in many different human organs (3). Previously, several research groups have revealed that SARS-CoV-1 patients have post-infection reproductive system complications (4, 5). Thus, in the present review, we demonstrate the molecular mechanism of the viral tropism, post-infected pathological features, and potentially detrimental effects in the male reproductive system in the COVID-19 patients. The review aims to draw appropriate conclusions about the impact of COVID-19 on male reproduction and put forward some new ideas for further research.

## The Molecular Mechanism of the Infection in the Male Reproductive System

In early 2020, it was demonstrated that the transmission mode of SARS-CoV-2 is mainly by droplets containing pathogens in the air (6). Droplets enter the nose and mouth and then carry pathogens to infect the upper respiratory tract. Firstly, SARS-CoV-2 entry into the host cells depends on the recognition of the angiotensin-converting enzyme 2 (ACE2) receptor (7). Since viral spike protein binds to the extracellular domain of ACE2, another critical factor, transmembrane protease serine protease-2 (TMPRSS2), mediates cleavage at the S1/S2 site of S protein, which is indispensable for viral membrane fusion (7). The viral RNA genome begins to replicate and instruct host-cell ribosomes to translate its structural proteins and polyproteins for viral capsid after SARS-CoV-2 enters the host cells (8). Viral particles are assembled in reticulum-Golgi intermediate compartment (ERGIC) (9) and subsequently are released by means of exocytosis for the spread of the infection (8).

ACE2 is regarded as the most essential key for viral entry; thus, expression patterns of ACE2 in different tissues and organs have become a focus in many research works, which may suggest potential routes of SARS-CoV-2 infection in humans. Previous studies have shown that ACE2 expression is abundant in the testis (10, 11). The proportion of ACE2-positive cells in the testis is more than that in the lung, which indicated that the testis might serve as a high-risk potential infection organ (12). Using single-cell RNA sequencing (scRNA-seq), it is reported that ACE2 is primarily expressed in Sertoli cells, which have the highest expression level, and then Leydig cells (LCs) and spermatogonia (13). It suggests a possible tropism of SARS-CoV-2 to the testis. The virus may infect Sertoli cells, disturbing the physiological process in which Sertoli cells control the germ cell environment by the secretion and transport of nutrients and regulatory factors, which is strongly related to spermatogenesis. Sertoli cells also play an important role in constituting the blood–testis barrier (BTB) (14). The BTB gives testes immune privilege separating autoantigens and host immune cells from germ cells and Sertoli cells. The Leydig cells are located in the interstitium of the testis where they are most vulnerable within the testis against the virus. LCs secrete androgen, which is indispensable in spermatogenesis and in maintaining secondary sex characteristics (15). Moreover, LCs also regulate testicular macrophage and lymphocyte numbers (16). Collectively, if SARS-CoV-2 infects these types of cells, the disruption of spermatogenesis will occur. In addition, a human sperm proteomic database reveals the presence of ACE2 in the human sperm while it is not detected through scRNA-seq (17). Furthermore, TMPRSS2 is expressed abundantly in prostasomes and is released into the seminal fluid from the prostate gland at ejaculation (18), which makes it possible for sperm to be vulnerable to SARS-CoV-2 infection.

Besides, the viral infection of cells requires cofactors, such as TMPRSS2 (19) and CD147 (20), to promote its invasion. TMPRSS2 is highly expressed in the kidney, epididymis, prostate, and seminal vesicles. TMPRSS2 expression was concentrated in spermatogonia and spermatids with relatively low levels in other cell types of testes (13). Stanley et al. used the scRNA-seq method to find that the proportion of co-expression of ACE2 and TMPRSS2 in testicular cells is less than 0.05% (21), which indicated that human testis is not susceptible to viral attack. However, transcript-level expression cannot represent the protein expression profiles of the human reproductive system. Additionally, most studies evaluated the risk of viral infection by examining the expression of ACE2 and TMPRSS2, which is sort of arbitrary, because other co-receptors promote the entry of SARS-CoV-2 in cells of the respiratory system. That is why the lung is the most susceptible organ to SARS-CoV-2, while single-cell sequencing data show that ACE2 expression was expressed in fewer than 0.1% of cells in the lung (22). Therefore, further studies are warranted to focus on protein expression of more viral receptors in testicular cells so that we could better elucidate the tropism of SARS-CoV-2 in the human reproduction system.

## The Impacts of COVID-19 on the Male Reproductive System

### Testicular Damage and Viral Transmission in the Semen of the COVID-19 Patients

Several studies focus on the reproductive pathology of COVID-19 patients. Ma et al. compared five COVID-19 patients with control patients and found that numerous degenerated germ cells (GCs) had sloughed into the lumen of seminiferous tubules (23). Two of five COVID-19 patients even showed symptoms similar to Sertoli cell-only syndrome. To ascertain the reason for the massive loss of GC, they found the significant presence of apoptotic cells and the infiltration of T lymphocytes, B lymphocytes, and macrophages in COVID-19 testes, which suggests that patients with COVID-19 had viral orchitis causing dysfunction of spermatogenesis. Yang et al. examined 12 postmortem testis samples of COVID-19 patients (24). They found that Sertoli cells showed swelling, vacuolation, and cytoplasmic rarefaction, and detachment from tubular basement membranes, and the number of Leydig cells was reduced, which is responsible for the decreased production of testosterone. Moreover, COVID-19 testes exhibited interstitial edema and mild lymphocytic inflammation corresponding to symptoms of orchitis. However, this study did not find significant alterations in spermatogenesis. Another research has reported damage of testes in autopsied testicular specimens consistent with autoimmune orchitis (25). Additionally, they also observed epididymitis in all cases. Two reports refer to the positive detection of SARS-CoV-2. Ma et al. have detected two testis samples of five positive for SARS-CoV-2 nucleic acid (23). Yao et al. examined 26 autopsy cases from deceased COVID-19 patients and found that SARS-CoV-2 spike existed in endothelia of the BTB, seminiferous tubules, and sperm in the epididymis in 3 of 26 cases (26). Yang et al. found only 1 case out of 10 was positive for the virus in the testis, whereas the positive one resulted from the blood with a high viral load rather than testicular tissue affection (24). However, the study of Song et al. showed that the SARS-CoV-2 is absent from both the semen and testis specimens of the COVID-19 patient (27). In addition, Peirouvi et al. observed an elevated level of pro-inflammatory cytokines, including TNF-α, IL-1b, and IL-6, and decreased expressions of genes related to BTB, claudin, occludin, and connexin-43 in testis tissues, which indicates that COVID-19 infection would disrupt BTB integrity (28). Collectively, SARS-CoV-2 is more inclined to affect the function of the testis by triggering autoimmune orchitis leading to the destruction of BTB. Whether testis injury is attributed to viral direct infection on testicular cells or uncontrolled autoimmune is still unclear. Testicular organoids may be a suitable platform for further research to investigate the molecular mechanism of viral impact on male reproduction.

There are several viruses reported that could cross the BTB and can be transmitted sexually, such as Zika (29) and HIV (30). Currently, the existence of SARS-CoV-2 in semen is controversial. It was reported that a semen test of 38 specimens found that 6 samples (15.8%) were positive for SARS-CoV-2, namely, 4 of 15 patients (26.7%) with the acute stage of infection and 2 of 23 patients (8.7%) recovering from COVID-19 (31). This result could be due to the fact that patients of the acute stage have a high blood viral load, which allows the virus to have more chance to reach the testes and enter the BTB mediated by local and/or systemic inflammation. Most studies suggested that there was no viral detection in the semen (23, 27, 32, 33). Considering the results of the aforementioned studies, it seems that in mild COVID-19 cases, the seminal presence of SARS-CoV-2 is unlikely or rare, while there is a lack of evidence to draw a firm conclusion in severe acute cases. Recently there are skeptical views on the positive results since the process of ejaculation and semen collection may be contaminated (34). Further data following the WHO guideline to avoid virus contamination is needed to clarify the issue (35). Nonetheless, in almost all cases, the sensitivity and specificity of the RT-PCR methods used to detect SARS-CoV-2 in seminal fluid have not been evaluated (36). It is not clear that current detection methods applied to nasopharyngeal swabs are properly available in viral detections in semen. Although most studies indicate a low risk of seminal infection, infection in semen and gametes remains a pending issue, which requires substantial epidemiological data concerning viral transmission from male recovered patients to previously unaffected sexual partners (34). In particular, it is noteworthy to evaluate the presence of SARS-CoV-2 for semen cryopreservation, because the virus can retain its biological pathogenicity in liquid nitrogen (37).

### Sperm Parameter Alterations in the COVID-19 Patients

The impact of SARS-CoV-2 on semen parameters has become an increasingly concerning issue to provide biological evidence for clinical recommendations in assisted reproduction centers. Holtmann et al. recruited 18 men, divided into mild and moderate groups, and a control group of 14 uninfected men (32). They examined sperm concentrations, the total number of sperm per ejaculate, the total number of progressive motility, and the total number of complete motility. Sperm concentrations, total progressive motility, and total sperm count of patients with moderate infection were significantly lower than the parameters of controls. However, there are limitations of this study in that the small sample can introduce sampling errors and it could not be compared with self-condition before infection. Li et al. observed that 9 out of 23 had decreased sperm concentration, representing the symptoms of oligozoospermia compared with the controls (25). Furthermore, all these patients with oligozoospermia have offspring, which demonstrated that they had intact fertility before infection. Segars et al. (38) indicated that male fertility may be severely affected for 72–90 days after infection due to decreased sperm concentration and motility (38). Furthermore, SARS-CoV-2, similar to other influenzas, could activate the cellular oxidative stress, leading to sperm DNA fragmentation, which is correlated to poor embryo development, lower implantation rate, and higher miscarriage rate (39–41). Febrility is one of the common symptoms of COVID-19 patients. The association of temporary alteration and sperm quality has been well studied (42). A fever can have significant effects on semen parameters and sperm DNA integrity, which suggests that a 3-month delay should be taken for COVID-19 male patients if they intend to have a conception or need an assisted reproductive techniques (ART) program. Furthermore, these adverse outcomes could be attributed to viral infections and may cause abnormal testosterone and LH levels and orchitis. To note, potential epigenome modifications of recovered patients’ gametes should be taken into consideration in future studies.

### Erectile Dysfunction in COVID-19 Patients

**Figure 1:**
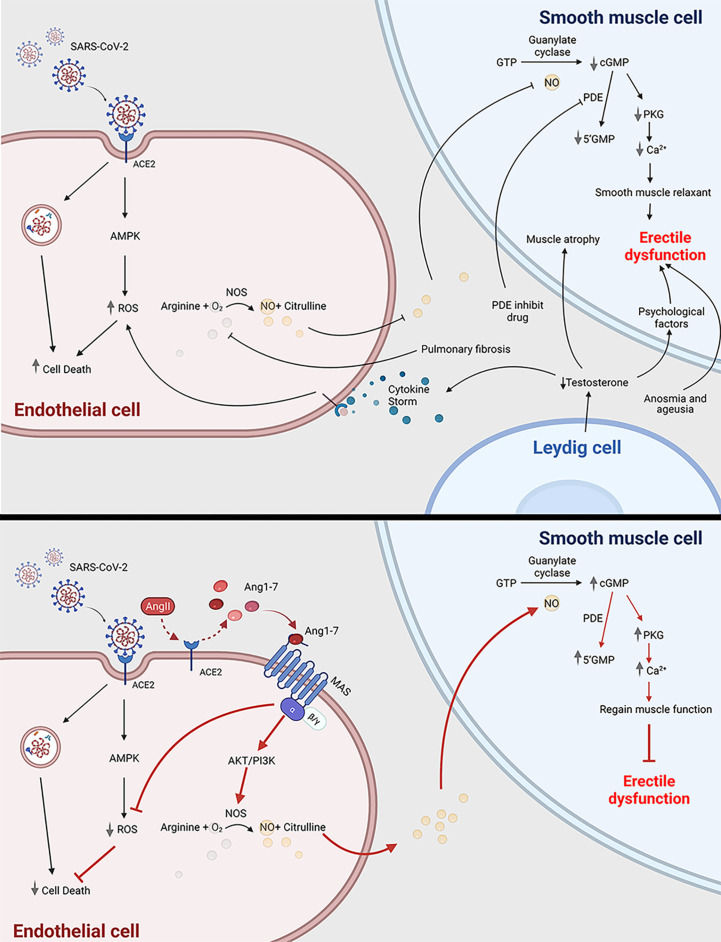
COVID-19 can cause erectile dysfunction. The potential mechanism of how COVID-19 infection is related to erectile dysfunction through impairing endothelial cell and smooth muscle cell (top panel); Ang1-7 is a potential novel drug target to treat erectile dysfunction in COVID-19 patients (bottom panel). ACE2, angiotensin-converting enzyme 2; SARS-CoV-2, severe acute respiratory syndrome coronavirus 2; ED, erectile dysfunction; ROS, reactive oxygen species; AMPK, AMP-activated protein kinase; Ang1-7, angiotensin 1-7; Ang II, angiotensin 2; PDE, phosphodiesterase; 5’GMP, Guanosine-5’-monophosphate; cGMP, cyclic guanosine monophosphate; PKG, Cyclic GMP–dependent protein kinase; NOS, nitric oxide synthase. This scheme was created using BioRender, accessed on Sept. 29, 2021.

Because of the high transmissibility of the infection and the higher severity rates among men than women (43, 44), there is a worry that erectile dysfunction (ED) is a possible consequence of COVID-19 for survivors. A preliminary study concluded that ED and COVID-19 seemed to be strongly associated after removing the possible bias resulting from age and BMI, factors that contribute to both increased prevalence of ED (45). The mechanisms may lie in the following aspects (**Figure 1**). Studies reported that most male participants with COVID-19 had decreased testosterone, suggesting hypogonadism (46, 47). Testis damage, including reduced Leydig cells, resulting in impaired steroidogenesis, may cause the hypogonadism in patients with COVID-19 (48). Low testosterone suppresses the expression of nitric oxide synthase and causes vascular smooth muscle cell atrophy (49). Furthermore, testosterone has an immunosuppressive function. Rastrelli et al. reported that the low testosterone in COVID-19 patients could predict poor prognosis and mortality (50). Hypogonadal patients have a higher level of TNF-α, IL-6, and IL-1β, which has a higher risk of vascular impairment (51). Therefore, the state of hypogonadism may play a crucial role in the onset of ED. Sexual activity is closely associated with psychological health. Psychological distress universally occurs and is affected by COVID-19. Due to the lockdown of many cities, the loss of freedom can lead to secondary losses such as losses of relationship, recreation, social support, and even income sources (52). The loss of relatives and friends can also trigger an increasing rate of depression. In addition, psychological factors resulting from low testosterone also contribute to ED (53). Endothelial dysfunction and cardiovascular impairment are other etiological factors. As the endothelium expresses ACE2 (54), SARS-CoV-2 is likely to affect the vascular endothelium including the penis during the systemic infection. Kresch et al. firstly reported the presence of SARS-COV-2 in the penis long after the initial infection in humans (55). On the other hand, the spike protein of the virus can alone damage endothelial cells by impairing the mitochondrial function, reducing eNOS activity and increasing ROS production through the deactivation of AMPK (56). The penile endothelial bed maintains vascular pressure, patency, and perfusion; inhibits thrombosis; and regulates the behavior of the neighboring vascular smooth muscle, all of which is crucial for erections (57). Additionally, erectile function is regarded as a predictor of heart disease (58, 59). ED can partially reflect the cardiovascular systemic state in COVID-19 patients. When the acute cytokine storm impairs their own organs, the cytokine storm may lead to the ROS-dependent apoptosis in vascular endothelial cells (60), which also contributes to the onset of ED. Another hypothesis that accounts for ED in the COVID-19 is decreased oxygen saturation as the result of pulmonary fibrosis hampers the availability of NO in the corpus cavernosum (61, 62). Another cause contributing to the potential onset of ED is anosmia and ageusia. Anosmia and ageusia, which play crucial roles in sexual activities, are symptoms of COVID-19 patients at the initial stage (63). The maintenance of penis erection requires both of them to trigger exciting messages to the brain (48, 64). PDE inhibitors are prevalent for drug treatment of ED because PDE inhibitors suppress degradation of cGMP, resulting in prolonged or enhanced erections (65). However, men with vascular-related diseases, such as diabetes, commonly do not respond well to PDE inhibitors (66). Novel approaches for ED treatment turn to novel drug target and stem cell therapy (67). The ACE2–Angiotensin-(1-7)–Mas axis may become an effective target to treat ED in COVID-19 patients. The mechanisms of this drug target is elucidated in **Figure 1**. Angiotensin 1-7 [Ang-(1-7)] is an endogenous heptapeptide from the renin–angiotensin system (RAS) with a cardioprotective role. Ang-(1-7) is proved to play an important role in eNOS activation and NO release by the PI3K/Akt pathway (68). Additionally, Ang-(1-7) also decreases the production of ROS (69), which may counteract the cytokine-induced dysfunction. In conclusion, further studies are needed to explore the pathogenesis of ED and investigate whether the occurrence of ED is just temporary or chronic.

## The Reproductive Toxicity of Drug Treatment of COVID-19

During the treatment of COVID-19, the use of antiviral drugs often neglects the potential reproductive toxicity. The following are the drugs that may be used/exploited for treatment referring to current guidelines (70). Remdesivir is approved by the FDA for the treatment of COVID-19 in hospitalized adult and pediatric patients (70). Up to now, there have been no reports of Remdesivir concerning the adverse effect on the human productive system despite one investigation suggesting the reproductive toxicity of Remdesivir, which was withdrawn by the authors for better experimental design (71). IFN-α is known for antiviral, antiproliferative, and immunoregulatory activities (72). de Lima Rosa et al. concluded that IFN-α within the normal dose range did not significantly influence sperm production, maturation, and motility, as well as levels of gonadal hormones (73). Ribavirin is used widely as an antiviral drug, which is a candidate for COVID-19 treatment due to the inhibition of viral RNA-dependent RNA polymerase (74). It is generally accepted that ribavirin exposure should be avoided during pregnancy due to the potential teratogen (75). Besides, the impact of ribavirin on spermatogenesis should be emphasized. Narayana et al. revealed that ribavirin significantly decreased the sperm count in a dose- and time-dependent pattern in rats. Additionally, ribavirin could cause a reversible decrease in sperm parameters including decreased sperm motility, DNA packaging abnormalities, and increase in sperm DNA fragmentation up to 8 months after drug discontinuation (76), which indicates that mandatory contraception should be taken after treatment discontinuation. Corticosteroids are used widely in the clinic for the reason that their potent anti-inflammatory effects might prevent or mitigate these deleterious effects. There is no evidence supporting adverse effects on human sperm, while corticosteroids possibly indirectly affect spermatogenesis and oocyte competence through the hypothalamic–pituitary–gonadal axis (77, 78). Broad-spectrum antibiotics are being widely used in patients with COVID-19 (79). Although most relevant studies are inconclusive, the potential reproductive toxicity of antibiotics should be considered (80). Hargreaves et al. found that amoxicillin impaired sperm viability at high doses (81). Another group demonstrated that therapeutic doses of penicillin G resulted in spermatogenic arrest in rats after treatment for 8 days (82). Moreover, high doses of nitrofurantoin can cause spermatogenic arrest, reduced sperm counts, and sperm immobilization, which is probably due to the failure of testicular cells to use carbohydrates and oxygen (83, 84). There are two drugs mispresented as “miracle drugs” by media misinformation and forged studies. However, both are not approved to treat COVID-19 patients. Chloroquine, an immunomodulant drug, is effective in reducing viral replication in SARS-CoV-2 infections, supported by *in vitro* data and clinical studies involving humans (85). Rat models showed that chloroquine reduces motility and fertilizing capacity of sperm (86, 87). However, there is a lack of sufficient data to make recommendations. Ivermectin has been shown to inhibit the replication of SARS-CoV-2 *in vitro* (88). Moreira et al. found a significant decrease in striatal dopaminergic system activity including dopamine release and lower testosterone levels in male rats, leading to a reduction in motor coordination (89). Above all, couples who have used related drugs are advised to seek professional advice and fertility check before planning to conceive.

##  Novel Model for Future Investigation of Fertility in COVID-19

Humanized ACE2 (hACE2) mice, which have overcome the natural insensitivity of mice to SARS-CoV-2 infection, are widely exploited for infection models and drug development. However, they are not an economical tool due to their high breeding fee and the lack of a qualified animal laboratory. Organoids are increasingly utilized for drug screening, toxicity assessment, and viral infection progression. Existing methods to evaluate the potential reproductive toxicity of drugs and viral infection require a large amount of animal sacrifice and could not ignore the individual differences. Organoids could provide more controllable, high-throughput, and faster evaluation techniques to simulate the microenvironment *in vivo*. In COVID-19, multiple research groups have resorted to organoid approaches to understand the tissue tropism of SARS-CoV-2. Penninger et al. established the capillary organoids and kidney organoids from human iPSCs and demonstrated that SARS-CoV-2 could directly infect cells in the capillary and kidney, which explains the spread of the virus throughout the body and the loss of kidney function in several severely infected cases (90). Another research group utilized human ASC-derived intestinal organoids to prove that SARS-CoV-2 could infect intestinal epithelium, the enterocyte, and replicate in intestines, suggesting that the intestine is a susceptible site of SARS-CoV-2 (91). To note, Lancaster and colleagues used brain organoids to investigate viral neurotropism and discovered that SARS-CoV-2 mainly infects the choroid plexus leading to damage to this brain barrier (92), and it should be emphasized that testis has a similar structure composed of cell–cell tight junctions. There is a restricted number of research groups that have reported and characterized human testicular organoids. Daniel and his colleagues developed the human testicular organoid to investigate the impact of ZIKV infection on testis (93). Until now, testicular organoids have not been used in studies about COVID-19 possibly because of difficulties of building human testicular organoids. Thus, further improvement of strategies to establish testicular organoids is needed. There is no denying that testicular organoid is a novel and efficient tool to investigate the susceptibility of testis to SARS-CoV-2 and to understand the alterations of post-infected reproductive capacity.

## Conclusion

It appears that COVID-19 influences different aspects of male reproduction including reproductive tracts, hormone, gametes, and sexual function. COVID-19 may trigger orchitis or epididymitis, thus impairing testis integrity and spermatogenesis. COVID-19 patients have decreased sperm concentration and motility. However, there seems to be a lack of consensus in the presence of SARS-COV-2 in semen and testis. Moreover, COVID-19 patients are exposed to a high risk of ED and thus we suggested Angiotensin-(1-7) as a novel drug target for ED in COVID-19. The current review also discusses the reproductive tract toxicity of drugs targeting COVID-19, and it also sheds new light on research on related fields and how the emerging model, such as the organoid, can be used to accelerate understanding of the related topic. Taken together, the impact of COVID-19 on the reproductive system still has outstanding unsolved questions. We need extensive clinical research and more efforts to investigate the long-term influence on male reproduction.

## Author Contributions

JG wrote the manuscript. KS performed the figure design and gave valuable advice. SW and HC contributed to collect relevant materials. All authors listed have made a substantial, direct, and intellectual contribution to the work, and approved it for publication.

## Funding

Our work was supported by National Key Research & Development grant (No. 2018YFC1003603) and the National Nature Science Fund of China (No. 81971445).

## Conflict of Interest

The authors declare that the research was conducted in the absence of any commercial or financial relationships that could be construed as a potential conflict of interest.

## Publisher’s Note

All claims expressed in this article are solely those of the authors and do not necessarily represent those of their affiliated organizations, or those of the publisher, the editors and the reviewers. Any product that may be evaluated in this article, or claim that may be made by its manufacturer, is not guaranteed or endorsed by the publisher.
